# The Preferred Retinal Locus for Reading in Central Vision Loss

**DOI:** 10.1167/iovs.66.14.12

**Published:** 2025-11-06

**Authors:** Arda Fidanci, Colin S. Flowers, Christian Larson, Stephen A. Engel, Gordon E. Legge

**Affiliations:** 1 Department of Psychology, University of Minnesota, Minneapolis, MN, United States; 2 Department of Ophthalmology and Visual Neurosciences, University of Minnesota, Minneapolis, MN, United States

**Keywords:** PRL, reading, central vision loss, eye movements, macular degeneration

## Abstract

**Purpose:**

People with central vision loss (CVL) often adopt a preferred retinal locus (PRL) for fixation outside the region of central loss. However, it is unknown how often the fixational PRL (fPRL) is used in reading. Here, we assessed if the reading PRL is in the same location as the fPRL.

**Methods:**

Participants read text on a computer screen. Reading was randomly interrupted by blanking the screen, and participants reported the word being read when the blanking occurred. The location of this word was compared to the fPRL location, measured with an eye tracker. If reading uses the fPRL, the two locations should overlap. We validated our method with 10 normally sighted controls for whom the fPRL and reading PRL should coincide at the fovea. We then tested 20 participants with CVL.

**Results:**

Control participants reported the word at the fPRL, or the next word, in 77.03% of trials. Participants with CVL reported the word at the fPRL, or the next word, on 70.45% of trials. Seventeen reported the word at the fPRL most frequently. Two reported the word to the right of the PRL most frequently, possibly due to a look-ahead strategy. One reported the word to the left of the fPRL most frequently, the target of many regressive saccades. Only one participant with CVL used a reading PRL remote from the fPRL, consistently reporting the word 2 degrees below the fPRL.

**Conclusions:**

Most of the participants with CVL in our sample used a reading PRL in close proximity to their fixation PRL.

The fovea is crucial for providing sharp vision and acts as an oculomotor reference in normal vision. However, macular diseases, such as age-related macular degeneration (AMD) and juvenile macular degeneration, can result in a scotoma, including the fovea, resulting in central vision loss (CVL). CVL can refer to a variety of patterns of loss, including absolute scotomas or relatively poor sensitivity in the central visual field, as well as sparing of foveal vision with ring-shaped or horseshoe-shaped central scotomas. Additionally, deficits in central vision associated with nystagmus are also important but are not included in our analysis. In the presence of a central scotoma, the visual system often adopts a specific retinal location on the retina outside the scotoma, known as the preferred retinal locus (PRL), to fixate on targets.^1^ The PRL is traditionally measured with simple fixation tasks; therefore, it is also known as the fixational PRL (fPRL).

Do people with CVL consistently process information using the same PRL across different tasks in daily life? It has been suggested that different retinal locations may be used, depending on stimulus properties such as brightness,^2^ size,^3^ and background illumination,^4^ or different visual tasks, including video watching^5^ and target-pointing.^6^

However, it remains unclear whether the fPRL is used in reading, which requires frequent saccadic eye movements. Past studies investigated the relationship between PRL and reading using scanning laser ophthalmoscopy. It has been claimed that people with CVL used different PRLs during reading tasks, including identifying isolated letters, word recognition, and paragraph reading, than in other tasks.^3^^,^^7^ Limitations of these studies include a lack of measurement of the fixation PRL and monocular eye tracking with the other eye covered, rather than natural binocular viewing.

In this study, we aimed to develop and validate a new eye tracker–based method to measure the retinal area engaged in reading. To validate our method, we recruited normally sighted young controls. We hypothesized that the fPRL coordinates and the location used for reading would overlap in normally sighted young controls since both correspond to the fovea. Next, we used the method in participants with CVL to test whether the retinal location used for reading corresponded to their fPRL. We expected that participants with CVL would rely on their fPRL for reading.

## Methods

### Participants

Ten normally sighted young adults (mean age = 21 years, SD = 3.16) from the University of Minnesota participated in the study. They were native English speakers with no known visual reading disabilities. All reported normal or corrected-to-normal binocular vision and had normal ocular health. Twenty participants with CVL were also recruited (age mean = 61.95 years, SD = 18.80). Exclusion criteria were peripheral vision loss and too poor acuity to execute reading. One additional participant was excluded because of failure in the eye-tracker calibration. An inclusion criterion was reporting at least some visual reading (with or without the help of magnification) in their daily lives.

All participants with CVL were native English speakers without known cognitive impairments, assessed by the Mini-Mental State Examination (score ≥25). All participants with CVL had functional reading vision and measurable acuity despite having heterogeneous eye diseases and varying levels of visual impairment. Eleven participants with CVL were diagnosed with AMD, 6 with Stargardt disease, and 3 with rod/cone dystrophy, cone dystrophy, and macular hole. The participants’ visual acuity measurements were obtained from their clinical records, measured with the Snellen acuity chart, and Snellen-based values were converted into logMAR values for the table. A summary of the visual characteristics of the participants is given in the Table. The study procedures were approved by the University of Minnesota institutional review board and followed the Declaration of Helsinki. Written informed consent was obtained from all participants before their participation.

**Table.** Demographics and Vision Characteristics of the Participants With CVL

Participant	Age, Y	Diagnosis	Distance VA (logMAR)^*^ OD–OS	Fixational Stability OD–OS	Scotoma Diameter OD–OS	Better Eye's PRL Location	MNREAD Reading Speed, wpm
CVL1	71	AMD	0.7–0.4	4.1 × 3.0–2.0 × 1.2	9 × 12–10 × 8	L	42
CVL2	79	AMD	2.3–0.7	7.2 × 11.4–9.1 × 6.9	7 × 10–4 × 4	R	36
CVL3	70	MH	0.5–0.6	3.4 × 5.4–3.8 × 3.6	2 × 4^†^–2 × 2	S, L	142
CVL4	67	AMD	1.09–0.17	10.9 × 8.8–3.5 × 4.2	8 × 8–4 × 2	S	129
CVL5	88	AMD	0.3–0.17	8.2 × 15.5–1.3 × 1.1	2 × 2–8 × 6	I, R	—^‡^
CVL6	77	AMD	0.47–0.79	5.4 × 4.2–4.1 × 2.6	4 × 1–4 × 6^†^	L	77
CVL7	54	AMD	0.1–0.17	2.4 × 2.3–2.5 × 2.9	12 × 6–6 × 8	S	108
CVL8	72	RCD	0.54–0.6	2.9 × 2.4–3.0 × 4.4	2 × 1–1 × 1	S	105
CVL9	53	Stargardt	—	9.1 × 5.9–6.7 × 5.7	10 × 10–8 × 6	I	45
CVL10	80	AMD	0.1–0.6	2.4 × 3.6–3.0 × 1.7	2 × 4–6 × 6	S, L	—^‡^
CVL11	49	Stargardt	0.39–0.87	5.2 × 7.3–4.1 × 6.9	4 × 8–6 × 10	I	83
CVL12	67	AMD	0.3–2.3	0.8 × 0.5–3.8 × 4.1	2 × 1–10 × 12	I,R	—^‡^
CVL13	81	AMD	0.3–0.47	6.3 × 3.7–2.1 × 1.8	1 × 1–2 × 1	I	92
CVL14	54	AMD	1.39–1.39	2.6 × 3.2–3.0 × 4.8	4 × 6–6 × 8	Central	98
CVL15	87	AMD	0.3–0.79	4.4 × 3.6–3.9 × 4.9	2 × 4–4 × 4	R	36
CVL16	24	Stargardt	0–0	1.9 × 2.5–1.6 × 1.4	2 × 2^†^–1 × 1	Foveal	126
CVL17	26	Stargardt	0.3–0.3	0.8 × 0.5–1.0 × 0.5	6 × 4^§^–6 × 1^§^	Foveal	113
CVL18	40	Cone dystrophy	0.6–0.6	3.5 × 7.6–4.1 × 3.8	2 × 1–2 × 1	S	85
CVL19	54	Stargardt	0–0	0.9 × 0.8–0.8 × 1.2	6 × 4^§^–6 × 4^§^	Foveal	153
CVL20	46	Stargardt	1–1	8.3 × 11.1–9.7 × 3.5	12 × 16–8 × 10	S, L	72
Mean	**61.95**						**90.71**
SD	**18.80**						**36.53**

Age, the individual diagnosis, visual acuity, fixational stability, scotoma diameter, reading speed (wpm), and PRL relative to scotoma. I, inferior; L, left; MH, macular hole; R, right; RCD, rod/cone dystrophy; S, superior; VA, visual acuity.

* Visual acuity measures were obtained from participants’ clinical records.

† Represents the area of relative scotoma, which is characterized by depressed light sensitivity (<15 dB).

‡ MNREAD reading-speed data unavailable due to technical issues.

§ Represents the horseshoe scotoma shape, allowing preserved foveal region.

### Apparatus

Stimuli were displayed on an Asus 24-inch LCD monitor operating at 144 Hz with a 1920 × 1080-pixel resolution. A desk-mounted Tobii Pro Spectrum (300 Hz; Tobii, Stockholm, Sweden) eye tracker recorded binocular eye positions. Participants’ heads were stabilized by using a chin rest. The experiment was presented and controlled in software using Tobii SDK packages and the Psychophysics Toolbox^8^ in MATLAB R2021a (MathWorks, Natick, MA, USA).

### Stimuli

Normally sighted control participants were shown 120 sentences, adapted from the MNREAD acuity test^9^ and generated using the MNREAD sentence generator.^10^ Each sentence was 60 characters long and formatted onto three lines.

The sentences were presented in black text (0.34 cd/m^2^) on a white background (231 cd/m^2^), at a viewing distance of 60 cm. For normally sighted controls, we used an Arial font with x-height = 0.78 (0.97 logMAR). Individual print sizes were determined for participants with CVL. An example sentence was presented in the print size with x-height = 0.5, and then the print size was gradually increased. Participants were asked to choose the smallest print that they could comfortably read. Their choice of comfortable print sizes was expected to be close to their critical print size values, as previously shown.^11^ The participants’ print sizes are given in Supplementary Table S1. Courier New was used as a font for participants with CVL since it provides better visual acuity for people with central vision loss.^12^ The line separation was 6.02 in normally sighted controls and ranged between 5.41 and 6.19 in participants with CVL, depending on their comfortable print size.

### MNREAD Reading Speed

An experimenter administered the MNREAD acuity test on the iPad. The maximum reading speed is provided in the Table.

### Microperimetry Measurement for Clinical Description

One author (CL), a trained optometrist, conducted microperimetry testing monocularly for both eyes, using the MAIA in participants with CVL. Participants were instructed to fixate a red circle with 1 diameter during microperimetry. As a measure of fixation stability, we used the bivariate contour ellipse area, provided by MAIA. Microperimetry testing was used to provide a clinical description of the participants, including scotoma information and PRL location relative to the scotoma.

The vision characteristics of participants are provided in the Table. Of particular note, three participants with CVL had fixation at or near the fovea based on MAIA testing: two of these participants had horseshoe-shaped scotomas with foveal sparing (CVL17 and CVL19). One participant (CVL16) had only reduced light sensitivity in the central region (for which we use the term *relative scotoma*) in the better eye, with a foveal fPRL. In addition to these participants, one participant (CVL14) had absolute scotomas in the central region in both eyes but, nevertheless, located the fixation target within the scotoma region. Another two participants with CVL (CVL3 and CVL6) had relative scotomas in the better eye, but their fPRLs were not located or close to the fovea. Although they are not absolute scotomas, the light sensitivity values are very low (<15 dB).

### Experimental Testing Procedure

At the beginning of each trial, the eye tracker was calibrated using a 5-point calibration task, with the participant fixating successively four fixation targets located in the corners of the screen and a fifth target at the center of the screen. The fixation targets were black-filled circles. We asked participants to look at each fixation point until it disappeared on the screen. The eye-tracker calibration was repeated until the deviation at each of the five fixation points met the Tobii criterion for accurate fixation.

Upon successfully calibrating the eye tracker, a sentence was presented on the display for reading while binocular gaze positions were recorded. The screen then blanked at an unpredictable time to interrupt the reading before the participant completed the sentence. For normally sighted controls, the screen was blanked randomly between 1.2 and 2.1 seconds, based on the approximate adult foveal reading speed of 250 words per minute (wpm).^13^^,^^14^

The time range for screen blanking was personalized for participants with CVL to account for differences in their reading speed. We asked them to read aloud 10 complete MNREAD sentences displayed on the screen before beginning the experiment. These sentences were presented at the comfortable print size chosen by the participants. The blanking range was set to between one-fourth and half of the average sentence reading time (Supplementary Table S1).

**Figure 1.**
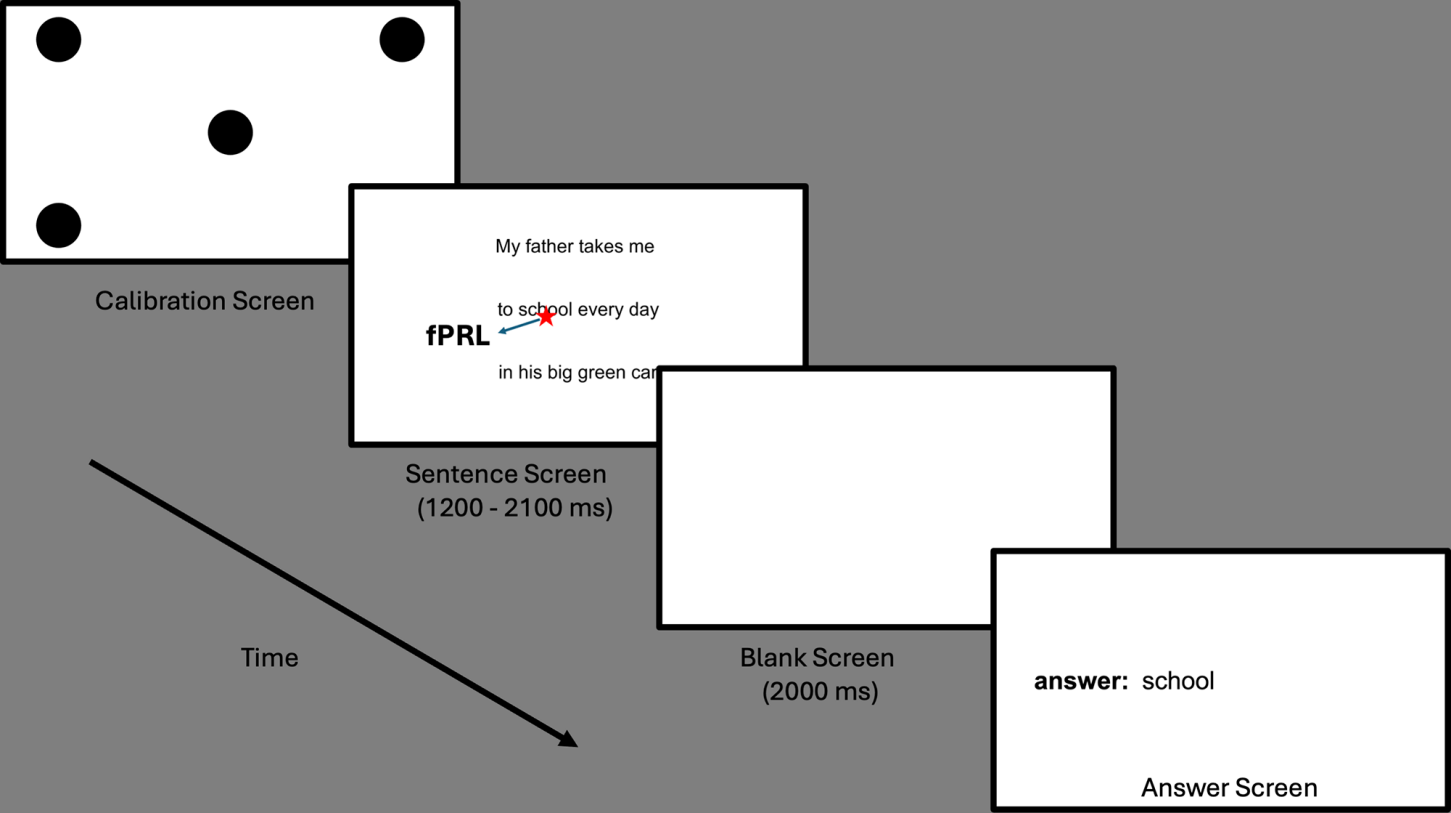
Experimental procedure. A 5-point calibration task was performed. Participants read text on the screen. The gaze position was captured at blanking time. Participants reported the word being read at the time of blanking.

For all participants, the blank screen was displayed for 2 seconds, and then a response prompt was displayed. Participants verbally reported the last word they read before the disappearance of the sentence, and the experimenter typed the reported word for each trial (see Fig. 1).

All participants completed the experiment binocularly. The task began with 10 practice trials and ended with 120 experimental trials for controls. The number of experimental trials for the participants with CVL varied individually since they had different reading speeds, which affected the duration of each trial. The participants completed as many trials as possible during the experimental session, with the number listed in Supplementary Table S1. Participants were encouraged to take breaks between trials as needed.

### Data Analysis

To quantify the relation between the fPRL (fovea in controls) and the word being read, we computed “word distance,” defined as the number of words between the fPRL and the retinal location of the reported words. While reading, the eye tracker recorded the x and y locations of the fPRL. The fPRL coordinates obtained at the blanking time of the reading screen were used to determine the word fixated, defined as the word at the fPRL position or closest to it. Specifically, we calculated the Euclidean distance in pixel units between the (x,y) coordinates of the fPRL and the bounding boxes of each word in the sentence, and we chose the one with the smallest distance as the fixated word. The “reported word” was defined as the word identified by the participant as the word being read when the interruption occurred.

**Figure 2.**
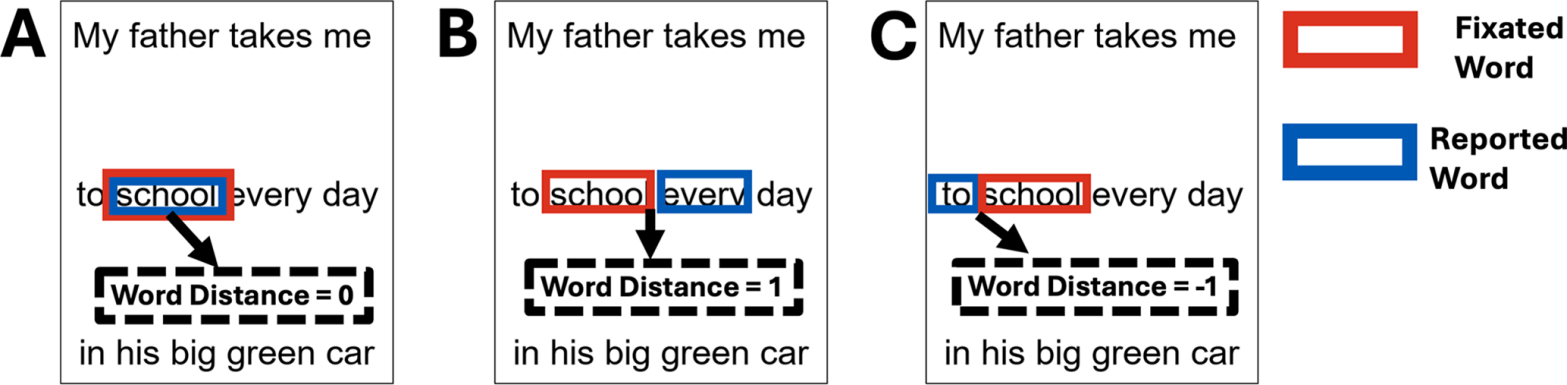
Word distance computation. The calculation was computed by subtracting the reported word's number from the fixated word's number. (**A**) Zero-word distance indicates reporting the word located at the fPRL. (**B**) Positive distances indicate reporting words located right of the fPRL. (**C**) Negative distances indicate reporting words left of the fPRL.

A zero-word distance indicated that the reported word was located at the fPRL (Fig. 2A), which would suggest that the fPRL is used for reading. Positive word distances indicated reporting words located right of the fPRL (Fig. 2B), and negative word distances indicated reporting words left of the fPRL (Fig. 2C). We excluded trials where an eye movement preceded the blanking by 80 ms or less (corresponding to less than 5% of trials) since it is unclear what word participants would report seeing last under such conditions.

We also compared the fPRL location to the reported word vertically. To do so, we computed the distance between the vertical position of the fPRL and the middle of the line of the text where the reported word was located. We first computed the pixel distance between fPRL coordinates and reported the word's vertical coordinates. Then, pixel distances were converted to degrees of visual angle.

## Results

### Validation of the Method With Normally Sighted Controls

**Figure 3.**
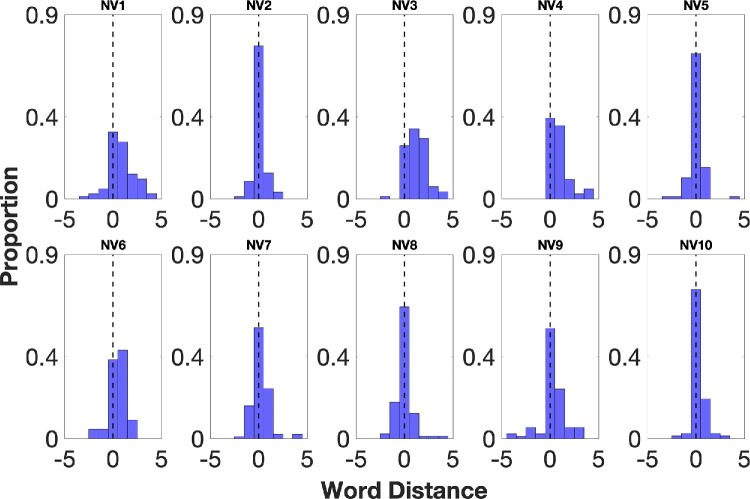
Individual word distance distributions for normally sighted controls. The proportion of trials is plotted as a function of word distance (the number of words between the reported word and the fPRL). The *black dashed* *line* at zero word distance indicates reporting the word at the fPRL. Eight of 10 controls reported the word located at the fPRL most frequently, shown in *blue*. Two controls (NV3 and NV6) reported one word right of the fPRL most frequently.

We validated the method by testing 10 normally sighted young controls. We recorded the word reported and the fPRL location when the screen blanked and calculated the distance between the two for each trial (see Methods). Figure 3 plots the distributions of these distances. Eight of 10 participants reported the word at the fPRL most commonly, in 58.53% ± 14.8% of trials. This pattern would be observed if the fPRL, which is expected to be the fovea in normal vision, is used in reading. In 20.39% ± 8.14% of trials, these participants reported one word located right of the fPRL, and they rarely reported words that were two or more words away from the right or left of the fPRL (<5%). The mean ± SD spread of the distribution was 0.94 ± 0.24, indicating a reasonably peaked distribution. The mean ± SD skew was 0.56 ± 0.80, indicating the relative commonness of reporting one or two words ahead of fixation compared to words behind fixation.

Two control participants (control 3 and control 6) reported one word to the right of the fPRL most frequently, in 38.21% ± 7.52% of trials. For these participants, the reported word was at the fPRL in 31.25% ± 10.71% of trials. These participants also reported the word that was two words to the right of the fPRL in 20.5% ± 15.67% of trials. Below, we discuss how “looking ahead” to read one or two words in advance of the fovea is a normal part of reading. Overall, across all control participants, in 77.03% of trials, the reported word was at the fPRL or one word to its right.

We also measured the vertical position of the fPRL during reading. In most trials, all control participants’ fPRL positions were close to the horizontal line through the midpoint of the line of text where the reported word was located, with a minimal offset (average median of the group = 0.19 ± 0.54, ranging between −0.71 and 0.92).

Overall, results from control participants indicate that the blanking method allows us to measure which word was being processed in reading and its spatial relation to fixation. As expected in most cases, the word reported as being read was at or near the fovea.

### Assessing Reading PRLs in Participants With CVL

**Figure 4.**
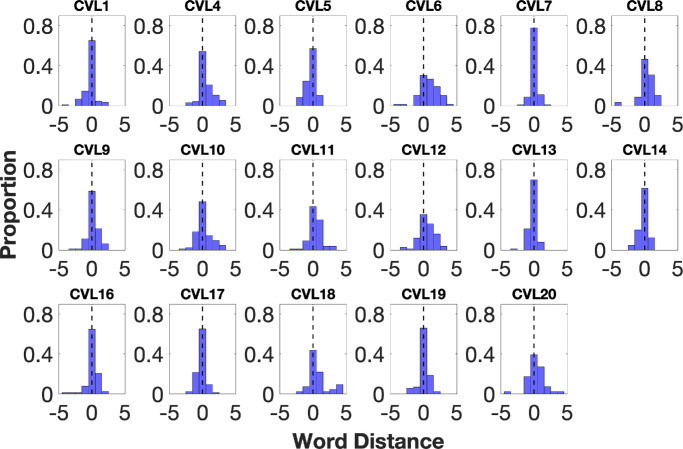
Individual word distance distributions for 17 participants with CVL. The proportion of trials is plotted as a function of word distance (the number of words between the reported word and the fPRL). The *black dashed* *line* at zero word distance indicates reporting the word at the fPRL.

We assessed the reading PRLs in 20 participants with CVL using the same methods. Figure 4 plots histograms of the distances between the fPRL and the reported word. Seventeen of the 20 participants with CVL reported the word at fPRL most frequently, in 54.43% ± 13.39% of trials on average. This pattern would be expected if the PRL used in reading is at or near the location used for fixation. For these 17 participants, one word to the right of the fPRL was the next most commonly reported, in 18.38% ± 8.21% of trials, followed by one word to the left of the fPRL, in 13.55% ±5.95% of trials. Reporting of words that were two or more words away from the right or left to the fPRL was rarely seen. The mean ± SD spread of the distribution was 0.99 ± 0.29, indicating a reasonably peaked distribution, similar to controls. The mean ± SD skew was −0.29 ± 0.71, indicating that most participants with CVL reported words on both sides of fixation, but a few extreme leftward outliers (−4 to −5 words) created a slight left tail—hence the negative skew.

**Figure 5.**
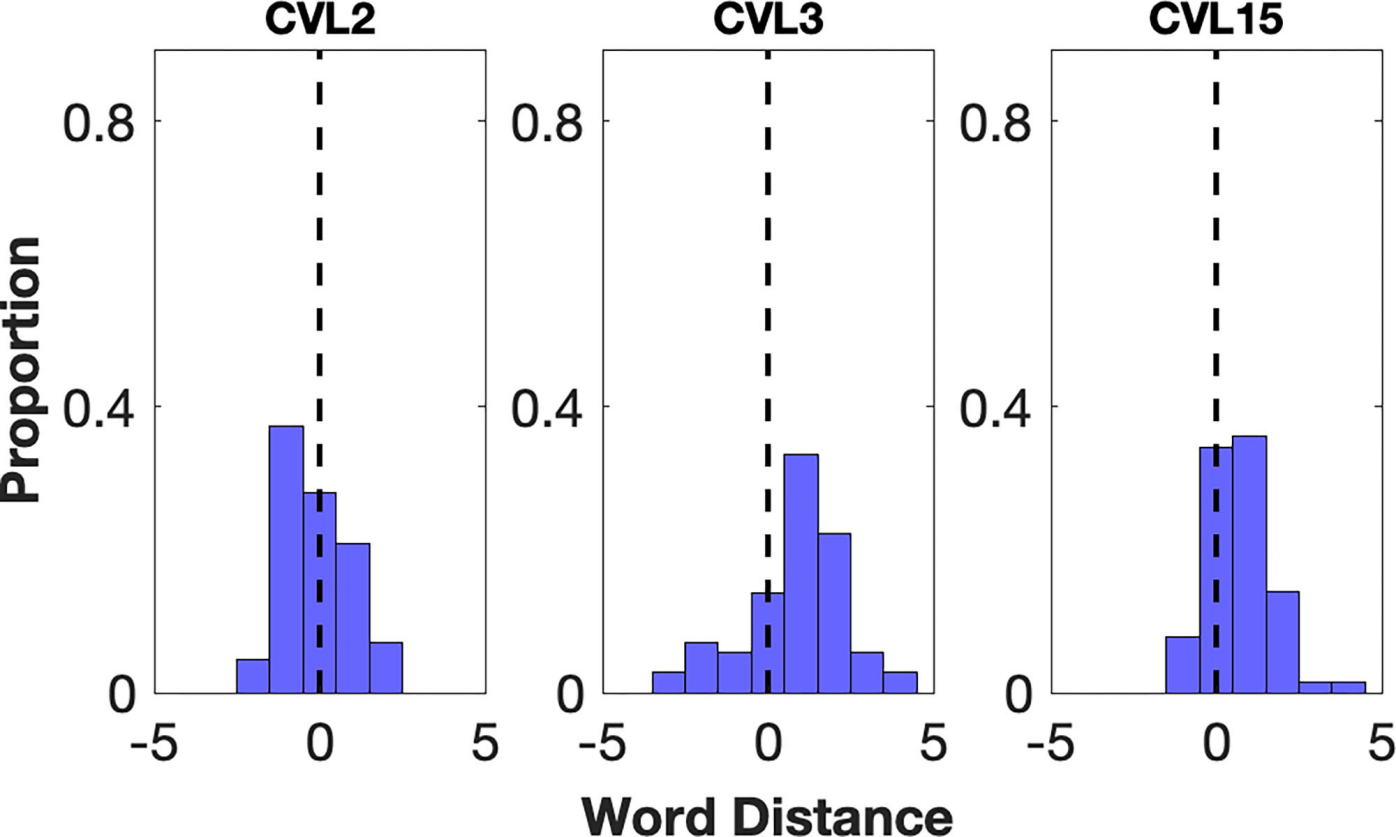
Individual word distance distributions for three participants with CVL (CVL2, CVL3, CVL15) whose distributions did not peak at 0.

Two participants with CVL (CVL3 and CVL15) reported one word to the right of the fPRL most frequently (33.33% and 35.94%, respectively) (Fig. 5). These two participants with CVL both had very high acuity in their better eye (0.5 and 0.3 logMAR, respectively) and small scotoma sizes. Their distributions were similar to two of the controls (control 3 and control 6). One participant with CVL (CVL2) showed a different pattern from all other participants with CVL and normally sighted controls. They reported one word to the left of the fPRL most frequently, in 37.21% of trials (Fig. 5). We will return to these unusual cases in the Discussion below. Overall, across all participants with CVL, in 70.45% of trials, the reported word was at the fPRL or one word to its right. This proportion is only slightly less than for the controls.

### Vertical Gaze Offset

**Figure 6.**
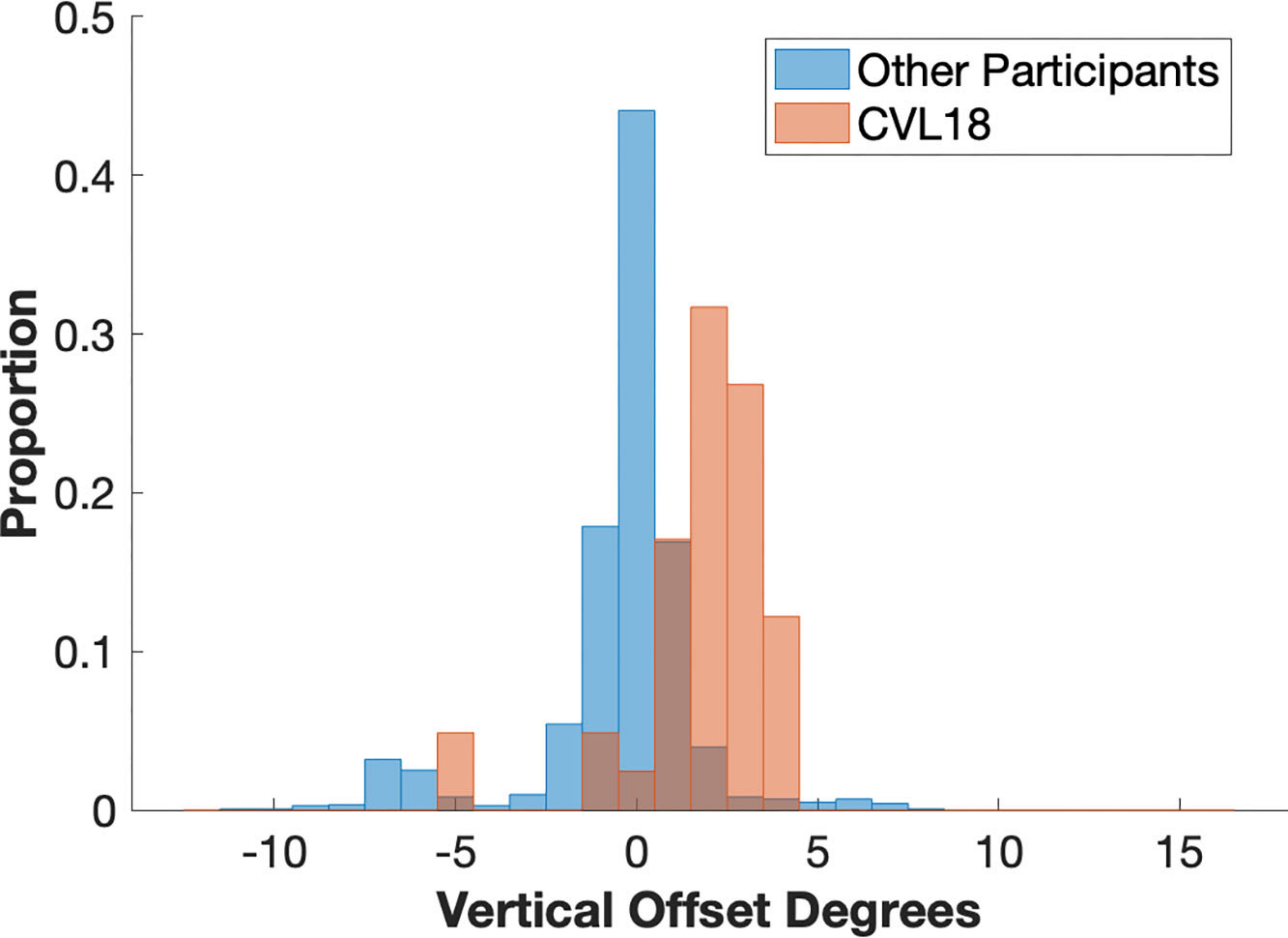
Histogram plot for the distributions of vertical gaze offsets between CVL18 and the other participants with CVL.

We also assessed the vertical offset of the fPRL in participants with CVL while reading. For 19 of the 20 participants with CVL, fPRLs were positioned close to the line of text where the reported word was located, with a minimal offset (average median of group = −0.11 ± 0.42, ranging between −0.89 and 0.57) (Fig. 6). However, CVL18 showed a systematic deviation of vertical gaze: the participant's fPRL was located on average 2.20 ± 2.01 above the horizontal line containing the reported word (Fig. 6). We will also discuss this case further in the Discussion.

In addition, all participants with CVL, including CVL18, showed a small peak (<5%) between −7 and −5 degrees in their vertical offset histograms (Fig. 6). These vertical offsets come from trials where participants reported the last word of the line “n,” and their gaze positions were located on the first word of the next line “n + 1.” In these cases, it is likely that the eyes had executed a return sweep to begin the next line, but the reader had not yet completed processing of the first word on the new line.

## Discussion

The reading PRL is a retinal area in the functioning periphery that is assumed to be used for reading. Determining whether it corresponds to the fPRL is important for understanding how people with CVL read. We have developed a new method for identifying the reading PRL relative to the fPRL.

The primary aim of the control study was to confirm and validate our method rather than to make a direct comparison between controls and participants with CVL. Results from normally sighted controls validated our method, with the word being read falling on the fovea or very close to it in most trials. In two control participants, the word being read was one word right of their fovea most frequently, corresponding to five to six characters. Normally sighted readers are able to recognize words within a seven- to eight-character span.^15^^–^^18^ Good readers may take advantage of this full span of letters and information from the sentence context to identify words to the right of the foveated word in reading. It is likely that these two control participants made better use of this “look-ahead” strategy.

Individual assessments of reading PRLs in participants with CVL demonstrated the predominant use of the fPRL in reading. In 16 of the 20 participants with CVL, the word being read was most frequently at their fPRL, with two participants showing offsets to the right, one to the left, and one a vertical offset. We will now address these four unusual cases.

CVL3 and CVL15 had word distance distributions that peaked one word to the right of the fPRL, similar to two of our normally sighted participants. These were two of the participants with CVL with high acuity in their better eye; the set of high-acuity participants with CVL might be expected to resemble our control participants, and so the pattern shown by these two is not surprising. CVL3 had the smallest scotoma sizes and the highest reading speed of participants with CVL (142 wpm). This makes it likely that they were simply using peripheral previewing in the same way that the normally sighted participants did. CVL15, who had larger scotomas and a slower reading speed, likely took advantage of context and visual span in order to guess the upcoming word. To test this, we analyzed the proportion of unmatched word trials, defined as words reported but not present in the sentence. CVL15 reported unmatched words in 17.1% of trials, which is two times higher than the CVL group average (7.89% ± 0.1%), likely reflecting dependence on this lexical inference strategy.^16^^,^^19^

CVL2’s word distance distribution peaked at one word to the left of the fPRL. This participant had frequent regressive saccades (30.52%) and a low reading speed (36 wpm). This pattern likely indicates that the processing of a word continued after the eyes shifted forward,^20^ rather than using a reading PRL distinct from the fPRL. On the other hand, it is possible that the participant used different PRLs for fixation and reading tasks, given that this participant likely had a large binocular scotoma. The slow reading speed and frequent regressive saccades might be consistent with a large binocular scotoma.

For CVL18, the word being read consistently showed a vertical offset from the fPRL. This unusual pattern indicates that they likely had a reading PRL different from their fPRL. Their microperimetry data showed that their reading PRL is located in an area with better light sensitivity (MAIA ∼25 dB) than the fPRL (MAIA ∼1 dB). The participant's fPRL is located in an area very close to the anatomic fovea. Accordingly, these results might be interpreted that the participant relied on their fovea for fixation, even when it was no longer very light sensitive, but had managed to find a better retinal area to use for reading. We used simple fixation dots in the calibration task. In a future study, a slightly different version of the calibration task in which dots are replaced with letters could be tested. This might provide further information on whether CVL18’s retinal location used for fixating letters would overlap with the reading PRL.

Our study has some limitations: one is that we tested reading with a fixed set of text characteristics, using only a single print size for each participant, brightness, spacing, and so on. These factors are known to influence reading, and it is possible that if they differed, they could shift the PRL location.^2^^,^^4^ Three of 20 participants with CVL had foveal fixation based on the MAIA results. For these, it may not be surprising that the fPRL corresponded closely to the reported words in reading. The remaining 17 participants with CVL had nonfoveal PRLs in their vision, based on the MAIA results, but still showed strong correspondence between the fPRL and the words reported in reading. It remains possible, however, that a larger sample of participants with large absolute central scotomas in both eyes would exhibit more offsets between the fPRL and the reading PRL. Our measurements comparing the fPRL to the reported word were made binocularly, enhancing the likelihood that our results apply to natural reading. However, our eye-tracker measurements do not specify the absolute retinal location of the binocular fPRL. It is likely the same as the monocular fPRL of the better eye as measured with MAIA (reported in the Table).^21^^,^^22^ Binocular scotoma mapping might provide more comprehensive information to characterize the vision characteristics and reading PRLs of participants under binocular viewing.^23^

In the future, using our method to determine the retinal location used for reading (whether it is fPRL or not) might be a helpful step in low-vision rehabilitation^24^ and in the design of promising assistive technologies, such as personalized spatial remapping of text.^25^

Spatial remapping is a method that aims to improve the effectiveness of residual vision of people with CVL by shifting information (e.g., letters during reading) from inside the scotoma to intact locations in the visual field via gaze-contingent displays.^25^^,^^26^ Determining the retinal location used for reading (whether it is fPRL or not) may be crucial to improving the effectiveness of remapping strategies by developing strategies that engage more with the reading PRL.

In summary, using our new and validated method to assess reading PRLs, we found that just one participant with CVL consistently showed a vertical offset from the fPRL, likely indicating a separate reading PRL from the fPRL. However, the remaining 19 of our 20 participants with CVL relied on a retinal location at or close to their fPRL in reading, suggesting the predominant use of the fPRL in reading tasks.

## Supplementary Material

Supplement 1
