# Supplementary material for: The Preferred Retinal Locus for Reading in Central Vision Loss
Source: Invest Ophthalmol Vis Sci. 2025 Nov 6;66(14):12. doi: 10.1167/iovs.66.14.12 (PMC12599517; doi:10.1167/iovs.66.14.12)
Supplement: Supplement 1 [file iovs-66-14-12_s001.docx]

| Participant | Trial Numbers | Mean Blanking Times in Seconds (± SD) | Print Size (x-height in degrees) | Print Size (logMAR) |
| --- | --- | --- | --- | --- |
| CVL1 | 89 | 6.22 (1.69) | 0.88 | 1.02 |
| CVL2 | 43 | 7.92 (2.25) | 0.86 | 1.01 |
| CVL3 | 72 | 1.65 (0.5) | 0.57 | 0.84 |
| CVL4 | 96 | 1.49 (0.46) | 0.6 | 0.86 |
| CVL5 | 49 | 2.89 (0.77) | 0.65 | 0.89 |
| CVL6 | 79 | 2.66 (0.67) | 0.68 | 0.91 |
| CVL7 | 90 | 1.8 (0.55) | 0.57 | 0.84 |
| CVL8 | 58 | 2.87 (0.94) | 0.62 | 0.87 |
| CVL9 | 80 | 3.08 (0.9) | 0.94 | 1.05 |
| CVL10 | 83 | 2.14 (0.65) | 0.62 | 0.87 |
| CVL11 | 106 | 3.21 (1.1) | 0.99 | 1.08 |
| CVL12 | 76 | 2.44 (0.74) | 0.57 | 0.84 |
| CVL13 | 65 | 2.71 (0.78) | 0.86 | 1.01 |
| CVL14 | 76 | 3.07 (0.8) | 1.38 | 1.22 |
| CVL15 | 64 | 5.32 (1.68) | 0.73 | 0.94 |
| CVL16 | 77 | 1.88 (0.49) | 0.75 | 0.95 |
| CVL17 | 83 | 2.23 (0.67) | 0.75 | 0.95 |
| CVL18 | 41 | 2.86 (0.78) | 0.7 | 0.92 |
| CVL19 | 85 | 1.74 (0.49) | 0.57 | 0.84 |
| CVL20 | 81 | 3.27 (0.97) | 0.91 | 1.04 |
| Mean | 74.65 | 3.07 | 0.76 | 0.95 |
| SD | 17.02 | 1.63 | 0.20 | 0.10 |

Supplementary Table 1. Participant Parameters: Trials, Blanking Timing, and Print Size.
